# Effectiveness and safety of anti-IL-5/Rα biologics in eosinophilic granulomatosis with polyangiitis: a two-year multicenter observational study

**DOI:** 10.3389/fimmu.2023.1204444

**Published:** 2023-06-30

**Authors:** Santi Nolasco, Andrea Portacci, Raffaele Campisi, Enrico Buonamico, Corrado Pelaia, Alida Benfante, Massimo Triggiani, Giuseppe Spadaro, Maria Filomena Caiaffa, Giulia Scioscia, Aikaterini Detoraki, Giuseppe Valenti, Francesco Papia, Alessandra Tomasello, Nunzio Crimi, Nicola Scichilone, Girolamo Pelaia, Giovanna Elisiana Carpagnano, Claudia Crimi

**Affiliations:** ^1^ Department of Clinical and Experimental Medicine, University of Catania, Catania, Italy; ^2^ Respiratory Medicine Unit, Policlinico “G. Rodolico-San Marco” University Hospital, Catania, Italy; ^3^ Institute of Respiratory Disease, Department of Translational Biomedicine and Neuroscience, University “Aldo Moro”, Bari, Italy; ^4^ Department of Health Sciences, University “Magna Graecia” of Catanzaro, Catanzaro, Italy; ^5^ Division of Respiratory Diseases, Department of Health Promotion Sciences, Maternal and Infant Care, Internal Medicine and Medical Specialties (PROMISE), University of Palermo, Palermo, Italy; ^6^ Division of Allergy and Clinical Immunology, University of Salerno, Salerno, Italy; ^7^ Center for Basic and Clinical Immunology Research (CISI), University of Naples Federico II, Naples, Italy; ^8^ Department of Medical and Surgical Sciences, School and Chair of Allergology and Clinical Immunology, University of Foggia, Foggia, Italy; ^9^ Department of Medical and Surgical Sciences, University of Foggia, Foggia, Italy; ^10^ Division of Internal Medicine and Clinical Immunology, Department of Internal Medicine and Clinical Complexity University of Naples Federico II, Naples, Italy; ^11^ Allergology and Pulmonology Unit, Provincial Outpatient Center of Palermo, Palermo, Italy

**Keywords:** EGPA, eosinophilic granulomatosis with polyangiitis, Churg-Strauss, mepolizumab, benralizumab, IL-5, remission, oral corticosteroid

## Abstract

**Background:**

Eosinophilic granulomatosis with polyangiitis (EGPA) is a rare vasculitis characterized by asthma, systemic manifestations, and blood and tissue eosinophilia.

**Objective:**

To assess the effectiveness and safety of mepolizumab (anti-IL-5) and benralizumab (anti-IL-5Rα) in EGPA for 24 months.

**Methods:**

We conducted a multicenter observational study, including patients with EGPA treated with anti-IL-5/Rα biologics in 9 Italian specialized facilities. Systemic disease activity, remission and relapse rate were evaluated from 3 to 24 months after treatment initiation. Respiratory outcomes, hematological parameters, corticosteroid (OCS) and immunosuppressants consumption were also assessed.

**Results:**

49 patients with relapsing-refractory EGPA were included [26 (53.1%) benralizumab 30mg, 20 (40.8%) mepolizumab 100mg, 3 (6.1%) mepolizumab 300mg]. Overall, 38.8% and 57.1% achieved remission after 12 and 24 months, respectively (69.2% benralizumab and 43.5% mepolizumab). Lower OCS intake and higher blood eosinophil count at baseline were associated with remission at 24 months. Both biologics exerted beneficial effects on severe asthma outcomes. Indeed, 61.2% (61.5% benralizumab and 60.8% mepolizumab) remained exacerbation-free during treatment. Lung function parameters showed improvements in the overall cohort (all *p<*0.05), but began to decline from month 12, especially with mepolizumab. Marked reduction in blood eosinophils was registered with mepolizumab (*p<*0.0001), while benralizumab depleted both eosinophils (*p<*0.0001) and basophils (*p*<0.0001). In general, 69.6% (76% benralizumab and 61.9% mepolizumab) of OCS-dependent patients lowered their daily dose by 75%, while 28.3% discontinued these drugs. Immunosuppressants were suspended in 88.2% of cases. Adverse events were reported in 8.2% of patients.

**Conclusions:**

These real-world data suggest that anti-IL-5/Rα biologics are effective and safe in the long-term as add-on treatments for patients with EGPA.

## Introduction

Eosinophilic granulomatosis with polyangiitis (EGPA) (1) is a small-to-medium vessel vasculitis characterized by asthma, often severe and difficult-to-treat, multi-organ involvement, along with blood and tissue eosinophilia (2, 3). Although rare, with an incidence of 0.5 to 4.2 cases per million per year (2), EGPA carries a significant burden due to frequent potentially life-threatening relapses (2, 4) and the need for prolonged steroid therapy, which exposes patients to glucocorticoid-related toxicity (5–7). According to EGPA guidelines, corticosteroids are currently the first-line treatment for remission induction and maintenance in patients with active disease, and in case of severe, life-threatening disease and/or major organ involvement, cyclophosphamide or rituximab should be added to glucocorticoids (8). Other traditional disease-modifying antirheumatic drugs (DMARDs), such as azathioprine or methotrexate, are routinely used in clinical practice for remission maintenance, despite the lack of evidence supporting their efficacy (6, 8). In this context, anti-IL-5/Rα biological therapies are appealing novel treatments for the management of EGPA (9–16).

Mepolizumab, a humanized monoclonal antibody (mAb) selectively targeting interleukin-5 (IL-5), a cytokine involved in eosinophils maturation, differentiation, recruitment, and survival, has proven to be safe and effective for the treatment of relapsing-refractory EGPA in the phase 3 MIRRA trial at the dosage of 300 mg (9). Nonetheless, real-world studies showed that even 100 mg, currently used for severe eosinophilic asthma, could be effective, particularly for respiratory manifestations (10–13).

Benralizumab also represents a promising drug, targeting the α subunit of the IL-5 receptor (IL-5Rα) expressed by eosinophils, prevents its interaction with IL-5 and stimulates their apoptosis through antibody-dependent cell-mediated cytotoxicity (ADCC) (17). Indeed, benralizumab showed to be very effective in patients with severe eosinophilic asthma (18–22), granting excellent steroid-sparing properties (23). However, as the phase 3 MANDARA trial (NCT04157348) (24) is still ongoing, limited evidence exists regarding benralizumab efficacy on EGPA (14–16).

In this multicenter real-world observational study, we aimed to evaluate the effectiveness and safety of a 24-month add-on treatment with anti-IL5/Rα mAbs in a cohort of patients with relapsing-refractory EGPA.

## Methods

### Study design

We conducted a multicenter, retrospective, observational study including patients with EGPA treated with benralizumab or mepolizumab between September 2017 and June 2022 at 9 Italian dedicated outpatients’ facilities, all part of the “Southern Italy Network on Severe Asthma Therapy”. A complete list of all the centers involved is available in this article’s **Supplementary Material**.

This study adhered to the Declaration of Helsinki and was approved by the “Catania 1” Ethics Committee of the Policlinico University Hospital (Approval Number 33/2020/PO - April 6, 2020, Catania, Italy). Written informed consent was obtained from all patients.

### Study population and treatment

Adult patients (≥18 years old), diagnosed with relapsing-refractory EGPA according to the American College of Rheumatology criteria (8, 25) despite maintenance therapy and severe eosinophilic asthma as defined by the European Respiratory Society/American Thoracic Society (ERS/ATS) guidelines (26), who received benralizumab (30 mg, once every 4 weeks for the first 3 doses, and then once every 8 weeks) or mepolizumab (100 mg every 4 weeks or 300 mg every 4 weeks) for 24 months as any line of treatment, were included. The indication for anti-IL-5/Rα biologics prescription adhered to the Italian eligibility policies as follow: 1) benralizumab required a confirmed diagnosis of severe eosinophilic asthma with a baseline peripheral blood eosinophil count ≥300 cells/μL, a minimum of 2 exacerbations in the prior year despite GINA step 5 treatment and/or maintenance oral corticosteroids (OCS); 2) mepolizumab 100 mg required a confirmed diagnosis of severe eosinophilic asthma, with a baseline blood eosinophil count ≥150 cells/μL with at least one value ≥300 cells/μL in the previous year, a minimum of 2 exacerbations in the prior year despite GINA step 5 treatment and/or maintenance OCS; 3) mepolizumab 300 mg was prescribed off-label for EGPA, as it was not yet approved in Italy at the time of data collection.

### Data collection

The shared data registry of the “Southern Italy Network on Severe Asthma Therapy”, created with the collaboration and final approval of all the participating centers was accessed for data collection. Demographic and clinical characteristics were gathered before biologics initiation (baseline) and at 3, 6, 12, and 24 months of follow-up.

Patients were evaluated by EGPA-dedicated multidisciplinary teams. Routine laboratory investigations, chest imaging, nasal endoscopy, echocardiography and abdominal ultrasonography were regularly performed (6, 8). In selected cases of organ-specific clinical manifestations, second level diagnostic procedures were carried out, including electromyography-electroneurography for peripheral neuropathy, gastrointestinal endoscopies for gastrointestinal symptoms, and cardiac MRI for clinical or echocardiographic signs of cardiomyopathy (6, 8).

The Birmingham Vasculitis Activity Score (BVAS) (27) and changes in organ manifestations, were used to assess the efficacy of anti-IL-5/Rα biologics in controlling overall systemic disease activity. The vascular damage index (VDI) (28) was calculated at the start of treatment to evaluate the resulting chronic injury induced by vasculitis. The revisited Five-Factor Score (29) was used to evaluate prognosis at diagnosis.

Remission was assessed as defined by 1) MIRRA (9) and MANDARA (24) trials [absence of disease activity (BVAS=0) and a dose of prednisolone or prednisone (or equivalent) of ≤4.0 mg/day]; 2) European League Against Rheumatism (EULAR) (30) [BVAS=0 and a dose of prednisolone or prednisone (or equivalent) ≤7.5 mg/day]. The proportion of patients in remission according to these criteria was also examined according to anti-neutrophil cytoplasmic antibodies (ANCA) positivity. Relapses were assessed in patients who had achieved remission and were defined by at least one of the following criteria (9, 24): 1) active vasculitis (BVAS>0) 2) worsening asthma and/or ear, nose, throat (ENT) manifestations leading to an increase in prednisolone or prednisone dose >4.0 mg/day and/or initiation of a new immunosuppressive therapy and/or hospitalization.

Severe asthma exacerbations were defined as disease worsening requiring ≥3 days of treatment with systemic corticosteroids (or a doubling of prednisolone equivalent dose if already on OCS) (31). Exacerbations treated with cycles of corticosteroids <7 days from each other were considered as the same exacerbation. Levels of asthma control were assessed using the asthma control test (ACT) (32, 33). Pulmonary function tests were performed following the ERS/ATS guidelines (34). Data on pre-bronchodilator forced expiratory volume in the first second (FEV_1_% and L), forced vital capacity (FVC%), FEV_1_/FVC%, and forced expiratory flow between 25% and 75% of FVC (FEF_25-75_%) were collected. Eosinophil and basophil count in peripheral blood were measured during treatment.

OCS dose changes (prednisone equivalent dose) and DMARDs discontinuation were also registered.

All adverse events occurred during treatment were also recorded, and their seriousness was assessed in accordance with the World Health Organization guideline (35).

Study outcomes were analyzed overall in the entire cohort and in patients’ subgroups receiving stable treatment either with benralizumab or mepolizumab.

### Statistical analysis

Data are presented as mean and standard deviation ( ± SD) for normally distributed continuous variables and as median and interquartile range (IQR) for continuous nonparametric variables. Categorical variables are stated as numbers (n) and percentages (%). The normality of data distribution was checked using the Anderson-Darling and Shapiro-Wilk tests. Unpaired Student *t*-test or Mann-Whitney test were used for comparison of continuous parametric and nonparametric variables at baseline. Mixed-effect model analysis, with Geisser-Greenhouse correction and Dunnett *post hoc* for repeated measures, were used to compare continuous outcomes from 3 to 24 months with time 0 (baseline). Fisher exact or McNemar tests were used for comparisons of categorical variables, when appropriate. Multiple logistic regressions (backward variable selection) were performed using variables trending towards significance (*p*<0.2) at univariate analyses, to determine factors independently associated with remission at month 12 and 24 with anti-IL-5/Rα biologics. The area under the receiver operating characteristic curve (AUC) and odds-ratios (OR) with 95% confidence intervals (95% CI) were calculated. Statistical analysis and figures were generated using Prism version 9.3.0 (GraphPad Software Inc., San Diego, California, USA) and SPSS Statistics 26 (IBM Corporation). A *p* value of <0.05 (2-sided) was considered statistically significant.

## Results

### Baseline patient characteristics

Among the 664 patients registered into the “Southern Italy Network on Severe Asthma Therapy” shared database, a cohort of 49 received anti-IL-5/Rα biologics [26 (53.1%) benralizumab 30 mg, 20 (40.8%) mepolizumab 100mg, 3 (6.1%) mepolizumab 300mg] for EGPA and were included in the analysis. A flow diagram of study participants is shown in **Figure E1 (Supplementary Material)**. The baseline characteristics are shown in **Table 1**.

**Table 1:** Baseline patients’ characteristics.

	All (n=49)	Benralizumab (n=26)	Mepolizumab (n=23)	p-value^*^
**Female, n (%)**	31 (63.3)	14 (53.8)	17 (73.9)	*0.2350*
**BMI, mean (SD)**	24.5 (3.5)	25.1 (2.4)	23.7 (4.4)	*0.1910*
**Age, years, mean (SD)**	50.3 (11.5)	49.2 (12.9)	51.5 (9.8)	*0.4922*
**Age at onset, years, mean (SD)**	37 (11.1)	38 (11.7)	35.8 (10.8)	*0.4963*
**Disease duration, years, mean (SD)**	12.8 (10.7)	10.9 (8.8)	14.9 (12.3)	*0.2058*
**Smoking status**				
Smoking history, n (%)	9 (18.4)	4 (15.4)	5 (21.7)	*0.7165*
Current smoker, n (%)	2 (4.1)	0 (0)	2 (8.7)	*0.2151*
**Patients with positive Skin Prick Tests, n (%)**	20 (40.8)	10 (38.5)	10 (43.5)	*0.7767*
**Active disease (BVAS >0), n (%)**	45 (91.8)	24 (92.3)	21 (91.3)	*0.9999*
**BVAS score, median (IQR)**	6 (4-10)	8 (4-10)	4 (2-10)	*0.1556*
**VDI, median (IQR)**	2 (1-2)	2 (2-4)	1 (0-2)	***0.0169***
**Five-Factor Score (revisited)**				
0, n (%)	29 (59.2)	17 (65.4)	12 (52.2)	*0.3944*
1, n (%)	16 (32.6)	7 (26.9)	9 (39.1)	*0.5424*
2, n (%)	4 (8.2)	2 (7.7)	2 (8.7)	*0.9999*
**Organ involvement**				
Constitutional, n (%)	11 (22.4)	5 (19.2)	6 (26.1)	*0.7341*
Arthropathy, n (%)	6 (12.2)	5 (19.2)	1 (4.3)	*0.1944*
Cutaneous, n (%)	9 (18.4)	4 (15.4)	5 (21.7)	*0.9999*
ENT, n (%)	37 (75.5)	23 (88.5)	14 (60.8)	***0.0128***
Pulmonary, n (%)	29 (59.2)	15 (57.7)	14 (60.8)	*0.9999*
Cardiac, n (%)	9 (18.4)	5 (19.2)	4 (17.4)	*0.9999*
Gastrointestinal, n (%)	7 (14.3)	4 (15.4)	3 (13)	*0.9999*
Renal, n (%)	3 (6.1)	2 (7.7)	1 (4.3)	*0.9999*
Peripheral neuropathy, n (%)	13 (26.5)	7 (26.9)	6 (26.1)	*0.9999*
**ANCA antibodies**				
ANCA positive, n (%)	13 (26.5)	7 (26.9)	6 (26.1)	*0.9999*
Perinuclear ANCA, n (%)	12 (24.5)	7 (26.9)	5 (21.7)	*0.5322*
MPO ANCA, n (%)	2 (4.1)	2 (7.7)	0 (0)	*0.4915*
PR3 ANCA, n (%)	1 (2)	0 (0)	1 (4.3)	*0.4694*
**Asthma exacerbations/year, median (IQR)**	4 (3-5.8)	4 (2.8-5)	4 (3-6.5)	*0.1301*
**ACT, median (IQR)**	13.5 (10-17)	15 (10.5-19.3)	12 (10-15.5)	*0.1462*
**FEV_1_, %, median (IQR)**	77 (59.3-89.5)	73 (58.5-86.8)	80.5 (59-91.3)	*0.4503*
**FEV_1_, L, median (IQR)**	2.3 (1.8-2.6)	2.3 (1.8-2.5)	2.3 (1.7-2.6)	*0.8176*
**FVC, %, median (IQR)**	90 (75-106)	89 (74.8-100)	90 (76.5-108.5)	*0.8279*
**FEV_1_/FVC, %, median (IQR)**	69 (60-75.5)	66.5 (59.8-73.3)	71 (62-78)	*0.2145*
**FEF_25-75_, %, median (IQR)**	41 (25-60)	35 (24.8-55)	57 (23.5-72.5)	*0.1321*
**FeNO, ppb, median (IQR)**	59.5 (30.3-126.5)	62.5 (39-136)	46.5 (19.3-112.8)	*0.2750*
**Laboratory parameters**				
Eosinophil counts in peripheral blood, cells/μL median (IQR)	775 (471-1565)	890 (506-1800)	705 (415-1409)	*0.5083*
Basophil counts in peripheral blood, cells/μL median (IQR)	60 (30-90)	70 (40-92)	30 (29-89)	*0.2500*
Neutrophil counts in peripheral blood, cells/μL median (IQR)	4705 (3725-6033)	5400 (4165-6543)	4030 (3498-6016)	*0.2391*
IgE, UI/ml, median (IQR)	154 (51-480)	158 (51-780)	133.5 (45.8-410)	*0.4882*
**Pharmacologic therapies**				
High dose ICS-LABA, n (%)	49 (100)	26 (100)	23 (100)	*0.9999*
LAMA, n (%)	29 (59.1)	14 (53.8)	15 (65.2)	*0.5618*
Patients on OCS, n, (%)	46 (93.9)	25 (96.1)	21 (91.3)	*0.5943*
OCS, mg/day, median (IQR)	10 (5-20)	10 (5-15)	12.5 (5-25)	*0.3345*
**Patients on DMARDs, n (%)**	17 (34.7)	11 (42.3)	6 (26.1)	*0.3675*
Azathioprine, n (%)	11 (22.4)	7 (26.9)	4 (17.4)	*0.5062*
Methotrexate, n (%)	4 (8.2)	3 (11.5)	1 (4.3)	*0.6119*
Cyclosporine, n (%)	1 (2)	0 (0)	1 (4.3)	*0.4694*
Rituximab, n (%)	1 (2)	1 (3.8)	0 (0)	*0.9999*
**Biologic therapy**				
Previous anti-IgE/anti-IL-5 mAbs, n (%)	1 (2)	1 (3.8)	0 (0)	*0.9999*

ACT, Asthma Control Test; ANCA, Anti-neutrophil cytoplasmic antibody; BMI, body mass index; BVAS, Birmingham Vasculitis Activity Score; DMARD, disease-modifying antirheumatic drug; ENT, ear, nose, and throat; FEF_25-75_, forced expiratory flow between 25% and 75% of FVC; FeNO, fractional exhaled nitric oxide; FEV_1_, forced expiratory volume in the 1st second; FVC, forced vital capacity; ICS-LABA, inhaled corticosteroids - long-acting beta-agonist; IgE, immunoglobulin-E; LAMA, long-acting muscarinic antagonist; mAb, monoclonal antibody; MPO, myeloperoxidase; OCS, oral corticosteroids (prednisone equivalent dose); PR3, proteinase 3; VDI, vascular damage index.

For normally distributed data, values are mean (standard deviation [SD]). For non-normally distributed variables, values are median (interquartile range [IQR]).

Bold entries highlight statistically significant *p*-values.

*Benralizumab vs mepolizumab.

The majority of patients were female (31 out of 49, 63.3%), with a mean age of 50.3 ± 11.5 years. Mean age at onset was 37 ± 11.1 years. Forty-five out of 49 (91.8%) had active disease at baseline. The most common manifestations were ENT (75.5%), pulmonary (59.2%), peripheral neurological (26.5%) and constitutional (22.4%) involvement. Thirteen (26.6%) were positive for ANCA antibodies. Before starting biological therapies, all patients received high-dose inhaled corticosteroids and long-acting beta-agonists; moreover, 46 (93.9%) were on OCS and 17 (34.7%) were on DMARDs. One patient (2%) received rituximab 8 months before benralizumab initiation.

Baseline demographic and clinical characteristics were comparable between the 2 groups of treatment, except for VDI score, which was higher in patients treated with benralizumab [2 (2-4) vs 1 (0-2), *p*=0.0169] and ENT manifestations, which were more frequent among patients who received benralizumab [23 (88.5%) vs 14 (60.8%), *p*=0.0128].

### Systemic manifestations and clinical remission

**Figure 1:**
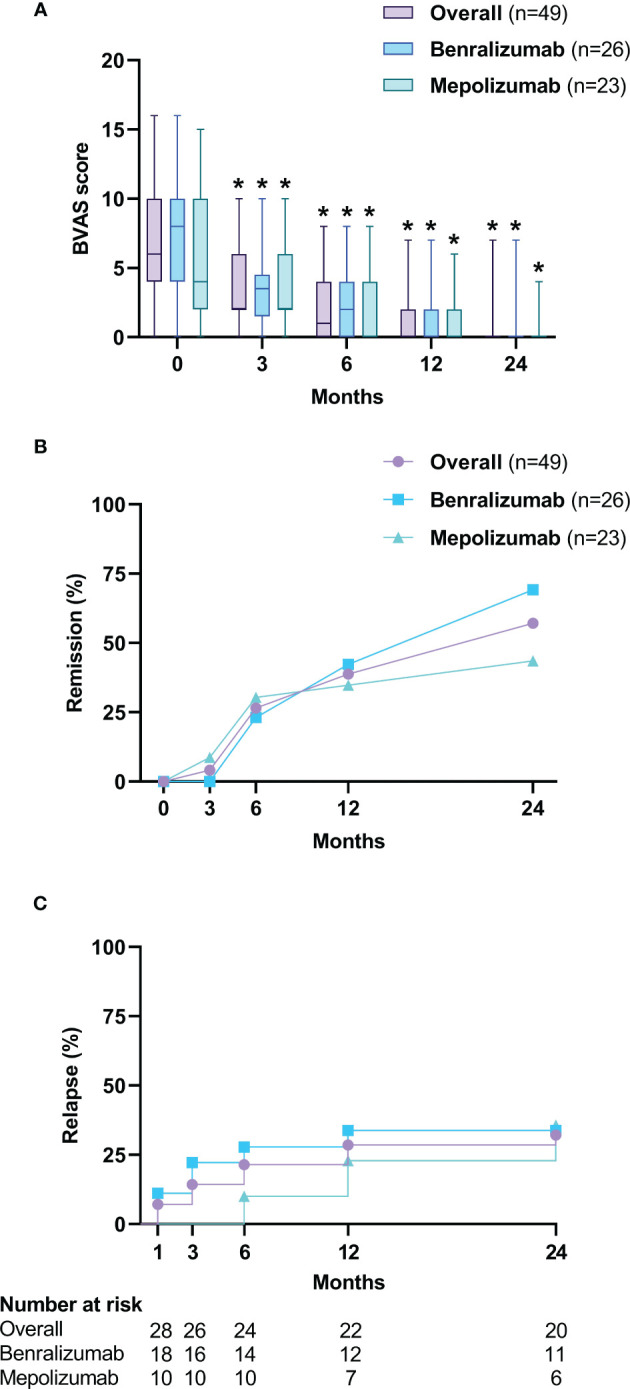
The effect of anti-IL-5/Rα biologicals on BVAS score is presented with box and whisker plots **(A)**. Boxes display median and interquartile ranges, whiskers define maximum and minimum values. **(B)** Percentage of patients in remission according to MIRRA criteria. **(C)** Kaplan-Meier curve for the occurrence of relapses. BVAS, Birmingham vasculitis activity score. *p<0.05.

Overall, we reported a progressive reduction in BVAS score during treatment (**Figure 1A**), with the median BVAS dropping from 6 (4-10) to 2 (2-6) (*p*<0.0001) already at 3 months, reaching 0 (0-2) at 12 months (*p*<0.0001), stably maintained at the 24 months follow-up [0 (0-0), *p*<0.0001]. The ability of benralizumab and mepolizumab to dampen EGPA systemic involvement is summarized in **Table E1 (Supplementary Material)**. A significant reduction in constitutional (from 22.4% to 2%, *p*=0.0044), arthropathy (from 12.2% to 0%, *p*=0.0412) and peripheral neurological manifestations (from 26.5% to 8.2%, *p*=0.0077) was already observed at 3 months, and maintained at 12 months, where a significant decreased in ENT (from 75.5% to 48.9%, *p*<0.0001) and pulmonary manifestations (from 59.2% to 38.8%, *p*<0.0001) was also registered.

At 3 months, clinical remission according to the MIRRA/MANDARA criteria was achieved in 4.1% of patients (2 out of 49, both on mepolizumab treatment) (**Figure 1B**), increased to 38.8% at 12 months and to 57.1% at 24 months, with a non-significative trend in favor of benralizumab [18 of 26 (69.2%) vs 10 of 23 (43.5%), *p*=0.0882] (**Table E2 in the Supplementary Material**). According to the less stringent EULAR criteria of clinical remission, 18.4% (9 of 49) achieved a complete response already after 3 months (**Figure E2 in the Supplementary Material**), with a gradual increase to 83.7% (41 patients of 49) after 24 months (**Table E2 in the Supplementary Material**). Notably, overall remission rates were similar in both ANCA+ and ANCA- patients, but at 3 months follow-up 50% (3 of 6) ANCA+ subjects achieved remission according to EULAR criteria, compared to 5.9% (1 of 17) of ANCA- (*p*=0.0353) (**Table E3 in the Supplementary Material**) in the mepolizumab group. Overall, among the 28 patients (57.1%) who achieved remission after 24 months of treatment [18 (69.2%) with benralizumab and 10 (43.5%) with mepolizumab], 9 (34.1%) experienced relapses. The proportion of patients with relapse was 33.3% (6 of 18) for benralizumab and 30% (3 of 10) for mepolizumab (**Figure 1C**).

### Baseline characteristics associated with remission

Patients who maintained clinical remission from 12 to 24 months follow-up were less OCS-dependent (82.3% vs 100%, *p*=0.0369) and were assuming lower dose of steroids [5 mg/day (2.5-10) vs 13.8 mg/day (6.3-25), *p*=0.0005] at baseline (**Table E4 in the Supplementary Material**). Multivariate logistic regression analysis (**Table 2**) showed that a lower OCS intake was independently associated with stable clinical remission from 12- to 24-months follow-up [OR 0.8 (0.7-0.93), *p*=0.0050].

**Table 2:** Baseline features independently associated with biologic-induced remission.

Model name	Baseline variables included	Final variables	*p-value*	Odds-ratio (95% CI)	AUC of model	Sensitivity	Specificity
**Stable remission from month 12 to 24** **vs** **No remission**	Disease duration Five-Factor Score (revisited) FEV_1_/FVC % OCS (mg/day) Patients on OCS	OCS (mg/day)	***0.0050***	0.8 (0.7-0.93)	0.711	74%	64%
**Remission at month 24** **vs** **No remission**	BMI Active smoke Constitutional symptoms Disease duration Active disease (BVAS >0) VDI Five-Factor Score (revisited) Cardiac involvement Peripheral neuropathy Blood eosinophil count Blood basophil count LAMA OCS (mg/day) Biologic therapy	OCS (mg/day) Blood eosinophil count	***0.0040*** ***0.0180***	0.8 (0.67-0.93) 1.002 (1.0-1.003)	0.844	88%	80%

ACT, asthma control test; BMI, body mass index; AUC, area under the receiver operating characteristic curve; BVAS, Birmingham vasculitis activity score; DMARD, disease-modifying antirheumatic drug; FEV_1_, forced expiratory volume in the 1st second; FVC, forced vital capacity; LAMA, long-acting muscarinic antagonist; OCS, oral corticosteroids (prednisone equivalent dose), VDI, vascular damage index.

Multiple logistic regression was performed (backward variable selection) using baseline variables trending towards significance (*p*<0.2).

Bold entries highlight statistically significant *p*-values.

Conversely, patients who achieved clinical remission up to 24 months had BVAS>0 (100% vs 81%, *p*=0.0282) (**Table E5 in the Supplementary Material**), less peripheral neurologic manifestations [14.3% vs 42.9%, *p*=0.0476), higher eosinophils count in peripheral blood [1005 eosinophils/μL (511-2295) vs 610 eosinophils/μL (434-1245), *p*=0.0189] and were assuming less OCS [5 mg/day (2.5-12.5) vs 15 mg/day (10-25), *p*=0.0043] at baseline (**Table E5 in the Supplementary Material**). Multivariate analysis found that lower OCS intake [OR 0.8 (95% CI 0.67-0.93), *p*=0.0040] and higher blood eosinophil count [OR 1.002 (95% CI 1.0-1.003), *p*=0.0180] were independently associated with remission at 24 months and predicted this outcome in 84.4% of cases (**Table 2**).

### Severe asthma outcomes and respiratory function

**Figure 2:**
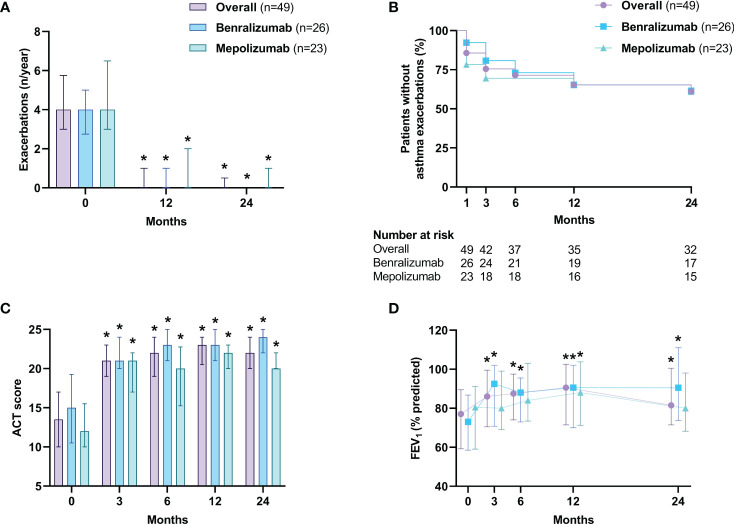
Effects of anti-IL-5/Rα biologicals on annual asthma exacerbation rate **(A)**, occurrence of asthma exacerbations **(B)**, ACT score **(C)** and FEV_1_% **(D)**. The annual asthma exacerbation rate, the ACT score and the FEV_1_% are expressed as median values (interquartile range). The occurrence of asthma exacerbations is presented with a Kaplan-Meier curve. ACT, asthma control test; FEV_1_, forced expiratory volume in the 1st second. *p<0.05.

**Figure 3:**
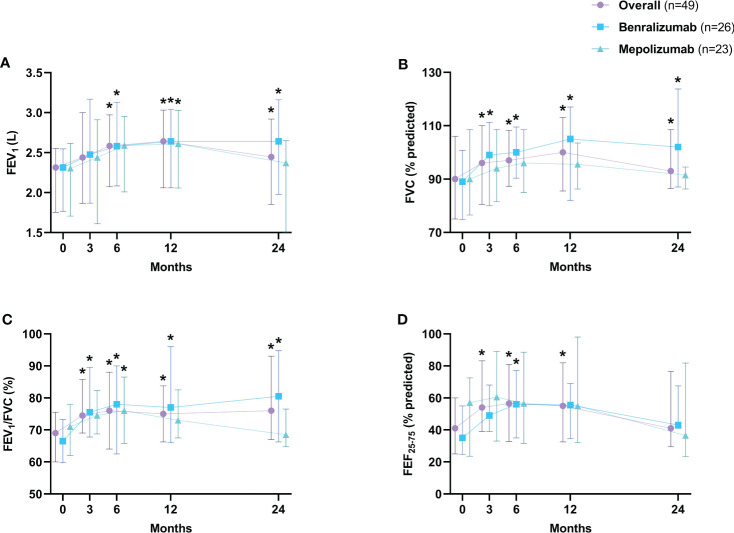
Changes in FEV_1_ (L) **(A)**, FVC% **(B)**, FEV_1_/FVC% **(C)** and FEF_25-75_% **(D)**. Data are expressed as median values (interquartile range). FEV_1_, forced expiratory volume in the 1st second; FVC, forced vital capacity; FEF_25-75_, forced expiratory flow between 25% and 75% of FVC. *p<0.05.

Asthma annual exacerbation rates dropped from 4 (3-5.8) to 0 (0-1) at 12 months (*p*<0.0001) and were consistently reduced to 0 (0-0.5) at 24 months (*p*<0.0001) (**Figure 2A**). In addition, 65.3% (32 of 49) of patients were exacerbation-free at 12 months and 61.2% (30 of 49) at 24 months, respectively (**Figure 2B**). Concerning asthma symptoms, the ACT score increased from 13.5 (10-17) to 21 (19-23) (*p*<0.0001) after 3 months, remaining above 20 during follow-ups, indicating good asthma control (*p*<0.0001 for both biologicals in each time point vs baseline) (**Figure 2C**). The pre-bronchodilator FEV_1_% went from 77% (59.3-89.5) to 90.6% (71.5-102.5) after 12 months (*p*<0.0001) with a slight decline to 81.5% (71.5-100.5) (*p*<0.0001) at month 24 (**Figure 2D**). Similarly, a statistically significant increase in FEV_1_ (L) (**Figure 3A**), FVC% (**Figure 3B**), FEV_1_/FVC% (**Figure 3C**) and FEF_25-75_% (**Figure 3D**), during the first 12 months of treatment and a progressive decline from month 12 to 24, especially for FEF_25-75_% (**Figure 3D**), were documented.

### Effect on blood eosinophil and basophil count

**Figure 4:**
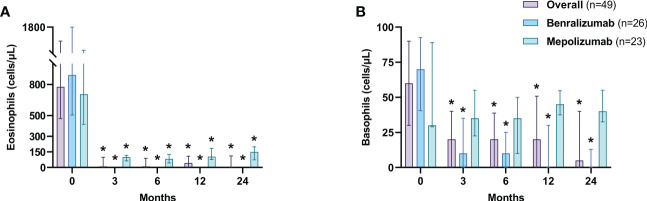
Changes in blood eosinophil **(A)** and basophil **(B)** count. Data are expressed as median values (interquartile range). *p<0.0001.

Eosinophils were depleted in patients who received benralizumab [0 cells/μL (0-0) at each time point (*p*<0.0001)], while in patients treated with mepolizumab, eosinophils lowered to 100 cells/μL (65-117) after 3 months (*p*<0.0001), 105 cells/μL (45-125) after 12 months (*p*<0.0001) and 150 cells/μL (74-200) after 24 months (*p*<0.0001) (**Figure 4A**). Treatment with benralizumab also resulted in a depletion of basophils after 12 months of treatment [from 70 cells/μL (40.5-92.5) to 0 cells/μL (0-30), *p*<0.0001], whereas in the mepolizumab group, basophils’ count remained similar to the baseline value (**Figure 4B**).

### Oral corticosteroid-sparing and discontinuation of disease-modifying anti-rheumatic drugs

**Figure 5:**
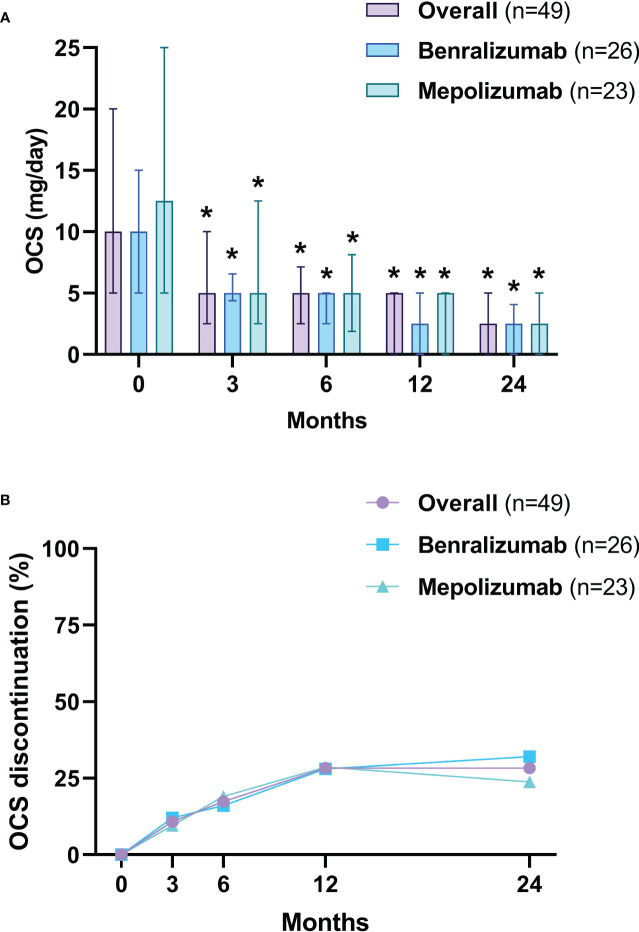
OCS daily dose reduction **(A)** and withdrawal **(B)**. The OCS daily dose is presented as median values (interquartile range). OCS, oral corticosteroids (prednisone equivalent dose). *p<0.05.

After 3 months, the median dose of OCS was reduced from 10 mg/day (5-20) to 5 mg/day (2.5-7.2) (*p*<0.0001), a further reduction to 2.5 mg/day (0.0-5) (*p*<0.0001) was observed at 24 months (**Figure 5A**). After 24 months, 69.6% (32 of 46) of OCS-dependent patients lowered their OCS daily dose ≥ 75% and 95.6% (44 of 46) achieved a daily dose ≤ 5 mg/day (**Table 3**). The proportion of patients able to discontinue OCS at 12 months was 28.3% (13 of 46) (**Figure 5B**). DMARDs were also suspended in 88.2% of patients (15 out of 17, *p*=0.0003) at 12 months (**Table E6 in the Supplementary Material**). At baseline, subjects on DMARDs had a higher VDI [3 (1.8-4.5) vs 1 (0-2), *p*=0.0268] and had a higher percentage of cardiac involvement (41.2% vs 6.3%, *p*=0.0051) (**Table E7 in the Supplementary Material**).

**Table 3:** Change in oral corticosteroid daily dose after 12 and 24 months of treatment.

	12 months			24 months		
Reduction from baseline in oral corticosteroids dose	Overall^*^	Benralizumab^†^	Mepolizumab^‡^	Overall^*^	Benralizumab^†^	Mepolizumab^‡^
> 0%, n (%)	37 (80.4)	21 (84)	16 (76.2)	39 (84.8)	24 (96)	15 (71.4)
≥ 50%, n (%)	36 (78.3)	21 (84)	15 (71.4)	38 (82.6)	25 (96)	14 (66.7)
≥ 75%, n (%)	27 (58.7)	15 (60)	12 (57.1)	32 (69.6)	19 (76)	13 (61.9)
100%, n (%)	13 (28.3)	7 (28)	6 (28.6)	13 (28.3)	8 (32)	5 (23.8)
Patients achieving a daily dosage ≤ 5 mg, n (%)	44 (95.6)	25 (100)	19 (90.4)	44 (95.6)	25 (100)	19 (90.4)
**Any increase or no change in dose, n (%)**	9 (19.6)	4 (16)	5 (23.8)	7 (15.2)	1 (4)	6 (28.6)

OCS, oral corticosteroids (prednisone equivalent dose).

^*^46 patients on OCS at baseline.

^†^25 patients on OCS at baseline.

^‡^21 patients on OCS at baseline.

### Safety

Four patients (8.2%) reported adverse events, 2 (7.7%) in the benralizumab group and 2 (8.7%) in the mepolizumab group. Most adverse events were considered mild-to-moderate [arrhythmia (n=1), urticaria (n=1), and otitis (n=1)], and only 1 (2%) experienced serious adverse events (pneumonia) in the benralizumab group (**Table E8 in the Supplementary Material**).

## Discussion

In this multicenter real-world study, we showed the effectiveness and safety of anti-IL-5/Rα biologics in relapsing-refractory EGPA with a relatively long follow-up period of 24 months. Our results indicate that both benralizumab and mepolizumab are effective in controlling the respiratory and systemic manifestations of EGPA with a good safety profile, exerting beneficial long-term effects and allowing for OCS/DMARD-sparing. To the best of our knowledge, this is the first real-world study exploring the clinical use of both biologics in the management of EGPA.

Most of our patients had active disease at baseline and were taking high-dose inhaled and systemic corticosteroids, which exposed them to develop steroid-related side effects. Treatment with anti-IL-5/Rα was associated with a decrease in disease activity and OCS consumption, allowing for clinical remission at 24 months in 57.1% and 83.7%, according to the MIRRA/MANDARA (9, 24) and EULAR (30) criteria, respectively.

Multivariable analysis of factors associated with clinical remission at 12 months showed that a lower OCS intake at baseline was independently associated with stable clinical remission from 12 to 24 months follow-up. Interestingly, remission at month 24 was associated with lower OCS intake at baseline and higher peripheral blood eosinophil counts. The latter might reflect the crucial role of eosinophils in the context of tissue damage and granulomatous inflammation in EGPA and since type 1 and type 2 inflammations frequently coexist in variable proportions (2, 36, 37), we might hypothesize that subjects with predominant type 2 inflammation and higher eosinophilia at baseline are the ideal phenotypes for anti-IL5/Rα biologics.

Anti-IL-5-targeted therapies represent a recent achievement in the treatment of patients with EGPA and research is growing in this field. Indeed, the phase 3 MIRRA trial (9) proved the superiority of subcutaneous mepolizumab 300 mg every 4 weeks versus placebo in achieving remission and lowering relapses. In addition, several observational studies have shown that even the 100 mg dosage used for severe eosinophilic asthma (38–42), could be effective (10–13). This is particularly important as the dosage tested in the registration study is not yet available in several countries. Indeed, although 88.5% of our mepolizumab cohort was treated with the 100 mg dose, the proportion of patients able to achieve clinical remission in our study (43% at 24 months) was similar to those reported in both the MIRRA trial (9) (32% at week 48) and in the largest real-world study on mepolizumab for EGPA (13) (31.2% and 37.9% at 12 months and 33.3% and 58.3% at 24 months for mepolizumab 100 mg and 300 mg, respectively).

For benralizumab, only a few case series and a small phase 2 open-label study have been published, showing its efficacy in improving systemic and respiratory symptoms while reducing OCS consumption (14–16). Nevertheless, benralizumab is currently used in clinical practice for the treatment of EGPA, mainly in centers with expertise in severe asthma, as shown in our cohort. Benralizumab stimulates eosinophils apoptosis through ADCC, with consequent depletion of both circulating and tissue cells (17). The efficient eosinophils depletion might explain similar clinical remission rates at 12 months compared with mepolizumab and a slight non-statistically significant trend in favor of benralizumab (69.2% vs 43.5%) at 24 months, respectively. Of note, neurological manifestations that seem to have both a vasculitic and neurotoxic etiology, driven by eosinophils’ products (43, 44), were reduced by 100% in the benralizumab group and by 50% in the mepolizumab group (45). However, most of our mepolizumab patients were taking the 100 mg dose licensed for asthma, and it is currently unknown whether the approved 300 mg dose offers greater benefits due to a more potent IL-5 blockade and eosinophils mitigation.

Lung function showed initial improvements in the overall cohort, with a slight decline from month 12, demonstrating stabilization of the effect of the disease on pulmonary capacity throughout the study period. Given the study duration (24 months) and age of these patients, a decrease in lung function over time might be part of the natural progression of the disease. Nevertheless, we cannot exclude that this decrease might have been related to a reduction in other maintenance therapies, especially OCS. Moreover, lung function parameters may only be partially associated with improvements in eosinophilic airway inflammation and the co-presence of mixed eosinophilic and neutrophilic inflammation in EGPA is associated with small airways disease severity and worse measures (46, 47). Interestingly, the decline in lung function was more pronounced with mepolizumab than with benralizumab. Since eosinophils are also responsible for the release of IL-13 and TGF-β, which promote sub-epithelial fibrosis (48), we can speculate that a more profound reduction of these cells in tissues may limit bronchial remodeling and, consequently, lung function decline.

Both biologics provided excellent steroid-sparing properties and allowed discontinuation of DMARDs while maintaining good control of respiratory and systemic manifestations, thereby reducing the risk of developing toxicity and adverse events from long-term treatment with OCS and DMARDs. Several mechanisms may have prevented the achievement of clinical remission in 42.9% of patients (56% and 31% for mepolizumab and benralizumab, respectively). EGPA is an immunologically heterogeneous disease; therefore, targeting eosinophils might provide limited benefits in those with a vasculitic phenotype. Furthermore, the already occurred tissue damage and fibrosis could have limited the effects of anti-eosinophils biologics in improving systemic manifestations. Lastly, exposure to long-term courses of OCS therapy could be responsible for adrenal insufficiency and jeopardize the withdrawal process and, subsequently, remission achievability.

Less than one in ten patients experienced adverse events, none of them requiring treatment discontinuation or hospitalization.

The main strength of our study is that it is based on real-world data and enabled us to include patients without the restrictive eligibility criteria of clinical trials, leading to a better understanding of the effectiveness of biological therapy in a more complex and heterogeneous EGPA population which is more representative of clinical practice. Indeed, data were collected by different severe asthma referral centers that are part of the “Southern Italy Network on Severe Asthma Therapy” whose aim is to provide consistent care for patients using a systematic and shared clinical approach, allowing for homogeneous cohort of patients and data collection. Moreover, we explored the long-term beneficial and adverse effects of both mepolizumab and benralizumab, particularly in case of complete eosinophil depletion.

This study has several limitations. The retrospective assessment of EGPA systemic disease activity and/or specific organ involvement might have limited the strength of our findings. The retrospective data collection may also be subject to missing data and information on the specific type of organ involvement or manifestations. Moreover, we cannot exclude that the heterogeneous distribution of the mepolizumab treatment regimens (100 mg or 300 mg) might have influenced its clinical efficacy. Moreover, these data are generated by routine clinical practice of severe asthma clinics; therefore, our EGPA cohort consisted of patients with severe eosinophilic asthma as their main respiratory manifestation, limiting the generalizability of our results to all real-life EGPA patients. Lastly, as with every retrospective observational study design, we cannot exclude that adverse events were underreported.

These data emphasize the role of anti-IL-5/Rα biologics in the effective control of respiratory and systemic EGPA manifestations and highlight the clinical effectiveness of targeting eosinophilic inflammation in these patients. However, further studies with larger sample size and longer observation periods are needed to evaluate the optimal application of anti-IL-5-targeted therapies, and head-to-head comparisons between the two biologics are desirable. The ongoing phase 3 MANDARA trial (NCT04157348) (24), comparing benralizumab 30 mg versus mepolizumab 300 mg, both administered subcutaneously every 4 weeks for EGPA will clarify the best option for patients.

## Data availability statement

The raw data supporting the conclusions of this article will be made available by the authors, without undue reservation.

## Ethics statement

This study was reviewed and approved by the “Catania 1” Ethics Committee of the Policlinico University Hospital (Approval Number 33/2020/PO - April 6, 2020, Catania, Italy). Patients/participants provided written informed consent.

## Author contributions

All authors contributed to data analysis, drafting or revising the article, have agreed on the journal to which the article will be submitted, gave final approval of the version to be published, and agree to be accountable for all aspects of the work.

## Southern Italy Network on Severe Asthma Therapy collaborators

**Vitaliano Nicola Quaranta**: Department of Translational Biomedicine and Neuroscience, University “Aldo Moro”, Bari, Italy; **Pietro Impellizzeri**: Department of Clinical and Experimental Medicine, University of Catania, Catania, Italy; **Rossella Intravaia**: Respiratory Medicine Unit, Policlinico “G. Rodolico-San Marco” University Hospital, Catania, Italy; **Morena Porto**: Department of Clinical and Experimental Medicine, University of Catania, Catania, Italy; **Elena Minenna**: Department of Medical and Surgical Sciences, School and Chair of Allergology and Clinical Immunology, University of Foggia, Italy; **Maria Pia Foschino Barbaro**, Department of Medical and Surgical Sciences, University of Foggia, Italy; **Alessia Lisotta**: Division of Respiratory Diseases, Department of Health Promotion Sciences, Maternal and Infant Care, Internal Medicine and Medical Specialties (PROMISE), University of Palermo, Palermo, Italy; **Dario Macaluso**: Division of Respiratory Diseases, Department of Health Promotion Sciences, Maternal and Infant Care, Internal Medicine and Medical Specialties (PROMISE), University of Palermo, Palermo, Italy; **Isabella Carrieri**: Division of Allergy and Clinical Immunology, University of Salerno, Italy; **Carla Messuri**: Center for Basic and Clinical Immunology Research (CISI), University of Naples Federico II, Naples, Italy; **Giuseppe Paglino**: Allergology and Pulmonology Unit, Provincial Outpatient Center of Palermo, Palermo, Italy.
