# Supplementary material for: Effectiveness and safety of anti-IL-5/Rα biologics in eosinophilic granulomatosis with polyangiitis: a two-year multicenter observational study
Source: Front Immunol. 2023 Jun 30;14:1204444. doi: 10.3389/fimmu.2023.1204444 (PMC10349177; doi:10.3389/fimmu.2023.1204444)
Supplement: Supplementary file 1 [file DataSheet_1.docx]

Supplementary Material

**Effectiveness and Safety of Anti-IL-5/Rα Biologics in Eosinophilic Granulomatosis with Polyangiitis: A Two-Year Multicenter Observational Study**

**Santi Nolasco^1,2†^, Andrea Portacci^3†^, Raffaele Campisi^2^, Enrico Buonamico^3^, Corrado Pelaia^4^, Alida Benfante^5^, Massimo Triggiani^6^, Giuseppe Spadaro^7^, Maria Filomena Caiaffa^8^, Giulia Scioscia^9^, Aikaterini Detoraki^10^, Giuseppe Valenti^11^, Francesco Papia^11^, Alessandra Tomasello^5^, Nunzio Crimi^1^, Nicola Scichilone^5^, Girolamo Pelaia^4^, Giovanna Elisiana Carpagnano^3*^, Claudia Crimi^1,2^ on behalf of the Southern Italy Network on Severe Asthma Therapy^§^**

^1^Department of Clinical and Experimental Medicine, University of Catania, Catania, Italy

^2^Respiratory Medicine Unit, Policlinico “G. Rodolico-San Marco” University Hospital, Catania, Italy

^3^Institute of Respiratory Disease, Department of Translational Biomedicine and Neuroscience, University “Aldo Moro”, Bari, Italy

^4^Department of Health Sciences, University “Magna Graecia” of Catanzaro, Catanzaro, Italy

^5^Division of Respiratory Diseases, Department of Health Promotion Sciences, Maternal and Infant Care, Internal Medicine and Medical Specialties (PROMISE), University of Palermo, Palermo, Italy

^6^Division of Allergy and Clinical Immunology, University of Salerno, Italy

^7^Center for Basic and Clinical Immunology Research (CISI), University of Naples Federico II, Naples, Italy

^8^Department of Medical and Surgical Sciences, School and Chair of Allergology and Clinical Immunology, University of Foggia, Italy

^9^Department of Medical and Surgical Sciences, University of Foggia, Italy

^10^Division of Internal medicine and Clinical Immunology, Department of Internal Medicine and Clinical Complexity University of Naples Federico II, Naples, Italy

^11^Allergology and Pulmonology Unit, Provincial Outpatient Center of Palermo, Palermo, Italy

† These authors share first authorship

*** Correspondence:**

Prof. Giovanna Elisiana Carpagnano, MD, PhD

Institute of Respiratory Disease, Department of Translational Biomedicine and Neuroscience, University “Aldo Moro”, Piazza Giulio Cesare 11, Bari 70124, Italy

Email: [elisiana.carpagnano@uniba.it](mailto:elisiana.carpagnano@uniba.it)

**^§^Southern Italy Network on Severe Asthma Therapy**

**Collaborators: Vitaliano Nicola Quaranta**: Department of Translational Biomedicine and Neuroscience, University “Aldo Moro”, Bari, Italy; **Pietro Impellizzeri**: Department of Clinical and Experimental Medicine, University of Catania, Catania, Italy; **Rossella Intravaia**: Respiratory Medicine Unit, Policlinico “G. Rodolico-San Marco” University Hospital, Catania, Italy; **Morena Porto**: Department of Clinical and Experimental Medicine, University of Catania, Catania, Italy; **Elena Minenna**: Department of Medical and Surgical Sciences, School and Chair of Allergology and Clinical Immunology, University of Foggia, Italy; **Maria Pia Foschino Barbaro**, Department of Medical and Surgical Sciences, University of Foggia, Italy; **Alessia Lisotta**: Division of Respiratory Diseases, Department of Health Promotion Sciences, Maternal and Infant Care, Internal Medicine and Medical Specialties (PROMISE), University of Palermo, Palermo, Italy; **Dario Macaluso**: Division of Respiratory Diseases, Department of Health Promotion Sciences, Maternal and Infant Care, Internal Medicine and Medical Specialties (PROMISE), University of Palermo, Palermo, Italy; **Isabella Carrieri**: Division of Allergy and Clinical Immunology, University of Salerno, Italy; **Carla Messuri**: Center for Basic and Clinical Immunology Research (CISI), University of Naples Federico II, Naples, Italy; **Giuseppe Paglino**: Allergology and Pulmonology Unit, Provincial Outpatient Center of Palermo, Palermo, Italy.

***“*SOUTHERN ITALY NETWORK ON SEVERE ASTHMA THERAPY”**

***List of the recruiting centers for this study***

1. Respiratory Medicine Unit - Policlinico “G. Rodolico-San Marco” University Hospital, Catania;
2. Respiratory Medicine Unit - A.O.U. Policlinico di Bari “Giovanni XXIII”, Bari;
3. Pulmonary Unit - A.O.U “Mater Domini”, Catanzaro;
4. Respiratory Medicine Unit - A.O.U. “Policlinico Giaccone”, Palermo;
5. Allergology and Clinical Immunology - A.O.U. “San Giovanni di Dio and Ruggi d'Aragona”, Salerno;
6. Allergology - A.O.U. “Federico II”, Naples;
7. Allergology and Clinical Immunology - University Hospital of Foggia, Foggia;
8. Institute of Respiratory Diseases - University Hospital of Foggia, Foggia;
9. Allergy and Pulmonary Medicine Unit - Center for Severe Asthma - ASP Palermo, Palermo.

**Figure E1.** Participants flow diagram.


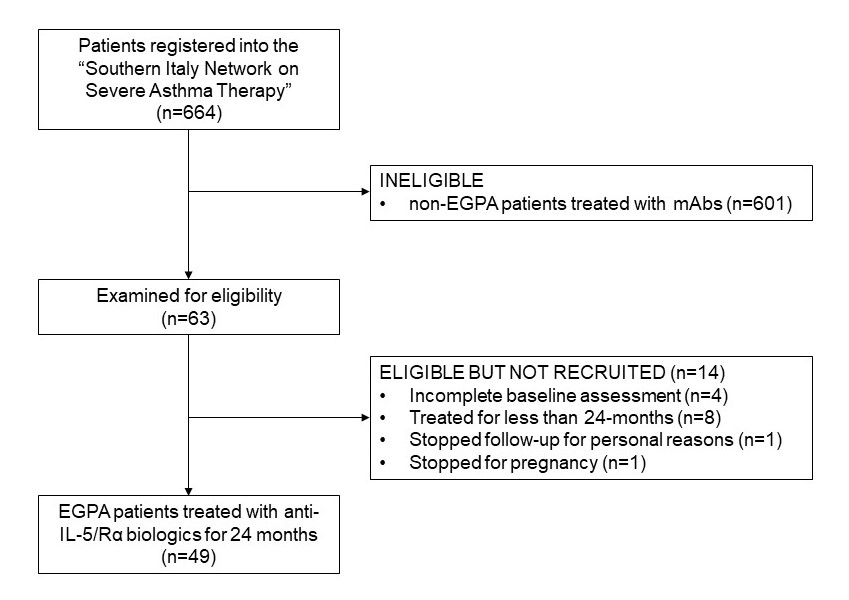


*Abbreviation: EGPA, eosinophilic granulomatosis with polyangiitis; mAbs, monoclonal antibodies.*

**Table E1.** Systemic manifestations during treatment

| **Organ involvement** | **Baseline** | **3 months** | ***p-value*** | **6 months** | ***p-value*** | **12 months** | ***p-value*** | **24 months** | ***p-value*** |
| --- | --- | --- | --- | --- | --- | --- | --- | --- | --- |
| **Constitutional** |  |  |  |  |  |  |  |  |  |
| Overall (n=49), n (%) | 11 (22.4) | 1 (2) | ***0.0044*** | 1 (2) | ***0.0044*** | 0 (0) | ***0.0026*** | 0 (0) | ***0.0026*** |
| Benralizumab (n=26), n (%) | 5 (19.2) | 0 (0) | *0.0736* | 0 (0) | *0.0736* | 0 (0) | *0.0736* | 0 (0) | *0.0736* |
| Mepolizumab (n=23), n (%) | 6 (26.1) | 1 (4.3) | *0.0736* | 1 (4.3) | *0.0736* | 0 (0) | ***0.0412*** | 0 (0) | ***0.0412*** |
| **Arthropathy** |  |  |  |  |  |  |  |  |  |
| Overall, n (%) | 6 (12.2) | 0 (0) | ***0.0412*** | 0 (0) | ***0.0412*** | 0 (0) | ***0.0412*** | 0 (0) | ***0.0412*** |
| Benralizumab, n (%) | 5 (19.2) | 0 (0) | *0.0736* | 0 (0) | *0.0736* | 0 (0) | *0.0736* | 0 (0) | *0.0736* |
| Mepolizumab, n (%) | 1 (4.3) | 0 (0) | *0.9999* | 0 (0) | *0.9999* | 0 (0) | *0.9999* | 0 (0) | *0.9999* |
| **Cutaneous** |  |  |  |  |  |  |  |  |  |
| Overall, n (%) | 9 (18.4) | 5 (10.2) | *0.1336* | 5 (10.2) | *0.1336* | 4 (8.2) | *0.0736* | 0 (0) | ***0.0077*** |
| Benralizumab, n (%) | 4 (15.4) | 2 (7.7) | *0.4795* | 2 (7.7) | *0.4795* | 2 (7.7) | *0.4795* | 0 (0) | *0.1336* |
| Mepolizumab, n (%) | 5 (21.7) | 3 (13) | *0.4795* | 3 (13) | *0.4795* | 2 (8.7) | *0.2482* | 0 (0) | *0.0736* |
| **ENT** |  |  |  |  |  |  |  |  |  |
| Overall, n (%) | 37 (75.5) | 34 (69.4) | *0.2482* | 30 (61.2) | ***0.0233*** | 24 (48.9) | ***<0.0001*** | 18 (36.7) | ***<0.0001*** |
| Benralizumab, n (%) | 23 (88.5) | 20 (76.9) | *0.2482* | 17 (65.4) | ***0.0412*** | 12 (46.1) | ***0.0026*** | 10 (38.5) | ***0.0009*** |
| Mepolizumab, n (%) | 14 (60.8) | 14 (60.8) | *0.9999* | 13 (56.5) | *0.9999* | 12 (52.2) | *0.4795* | 8 (39.1) | ***0.0412*** |
| **Pulmonary** |  |  |  |  |  |  |  |  |  |
| Overall, n (%) | 29 (59.2) | 24 (49) | *0.0736* | 21 (42.8) | ***0.0133*** | 19 (38.8) | ***0.0044*** | 10 (20.4) | ***<0.0001*** |
| Benralizumab, n (%) | 15 (57.7) | 12 (46.1) | *0.2482* | 10 (38.5) | *0.0736* | 10 (38.5) | *0.0736* | 6 (23.1) | ***0.0077*** |
| Mepolizumab, n (%) | 14 (60.8) | 12 (52.2) | *0.4795* | 11 (47.8) | *0.2482* | 9 (39.1) | *0.0736* | 4 (17.4) | ***0.0044*** |
| **Cardiac** |  |  |  |  |  |  |  |  |  |
| Overall, n (%) | 9 (18.4) | 7 (14.3) | *0.4795* | 6 (12.2) | *0.2482* | 4 (8.2) | *0.0736* | 3 (6.1) | ***0.0412*** |
| Benralizumab, n (%) | 5 (19.2) | 4 (15.4) | *0.9999* | 3 (11.5) | *0.4795* | 2 (7.7) | *0.2482* | 1 (3.8) | *0.1336* |
| Mepolizumab, n (%) | 4 (17.4) | 3 (13) | *0.9999* | 3 (13) | *0.9999* | 2 (8.7) | *0.4795* | 2 (8.7) | *0.4795* |
| **Gastrointestinal** |  |  |  |  |  |  |  |  |  |
| Overall, n (%) | 7 (14.3) | 5 (10.2) | *0.4795* | 5 (10.2) | *0.4795* | 4 (8.2) | *0.2482* | 1 (2) | ***0.0412*** |
| Benralizumab, n (%) | 4 (15.4) | 2 (7.7) | *0.4795* | 2 (7.7) | *0.4795* | 2 (7.7) | *0.4795* | 1 (3.8) | *0.2482* |
| Mepolizumab, n (%) | 3 (13) | 3 (13) | *0.9999* | 3 (13) | *0.9999* | 2 (8.7) | *0.9999* | 0 (0) | *0.2482* |
| **Renal** |  |  |  |  |  |  |  |  |  |
| Overall, n (%) | 3 (6.1) | 2 (4.1) | *0.9999* | 1 (2) | *0.9999* | 0 (0) | *0.2482* | 0 (0) | *0.2482* |
| Benralizumab, n (%) | 2 (7.7) | 1 (3.8) | *0.9999* | 0 (0) | *0.4795* | 0 (0) | *0.4795* | 0 (0) | *0.4795* |
| Mepolizumab, n (%) | 1 (4.3) | 1 (4.3) | *0.9999* | 1 (4.3) | *0.9999* | 0 (0) | *0.9999* | 0 (0) | *0.9999* |
| **Peripheral neuropathy** |  |  |  |  |  |  |  |  |  |
| Overall, n (%) | 13 (26.5) | 4 (8.2) | ***0.0077*** | 4 (8.2) | ***0.0077*** | 3 (6.1) | ***0.0044*** | 2 (4.1) | ***0.0026*** |
| Benralizumab, n (%) | 7 (26.9) | 1 (3.8) | ***0.0412*** | 1 (3.8) | ***0.0412*** | 0 (0) | ***0.0233*** | 0 (0) | ***0.0233*** |
| Mepolizumab, n (%) | 6 (26.1) | 3 (13) | *0.2482* | 3 (13) | *0.2482* | 3 (13) | *0.2482* | 2 (8.7) | *0.1336* |

*Abbreviation: ENT, ear, nose, and throat.*

Changes in systemic manifestations during follow-ups were assessed using the McNemar test.

Bold entries highlight statistically significant *p*-values.

| **Type of response** | **3 months** | **6 months** | **12 months** | **24 months** |
| --- | --- | --- | --- | --- |
| **MIRRA/MANDARA remission criteria (BVAS = 0 and OCS ≤ 4 mg/day)** |  |  |  |  |
| Overall (n=49), n (%) | 2 (4.1) | 13 (26.5) | 19 (38.8) | 28 (57.1) |
| Benralizumab (n=26), n (%) | 0 (0.0) | 6 (23.1) | 11 (42.3) | 18 (69.2) |
| Mepolizumab (n=23), n (%) | 2 (8.7) | 7 (30.4) | 8 (34.8) | 10 (43.5) |
| **EULAR remission criteria (BVAS = 0 and OCS ≤ 7.5 mg/day)** |  |  |  |  |
| Overall (n=49), n (%) | 9 (18.4) | 23 (46.9) | 32 (65.3) | 41 (83.7) |
| Benralizumab (n=26), n (%) | 5 (19.2) | 13 (50.0) | 18 (69.2) | 23 (88.5) |
| Mepolizumab (n=23), n (%) | 4 (17.4) | 10 (43.5) | 14 (60.9) | 18 (78.3) |

**Table E2.** Remission criteria

*Abbreviation: BVAS, Birmingham Vasculitis Activity Score, OCS, oral corticosteroids (prednisone equivalent dose).*

**Figure E2.** Percentage of patients in remission according to EULAR criteria.

*
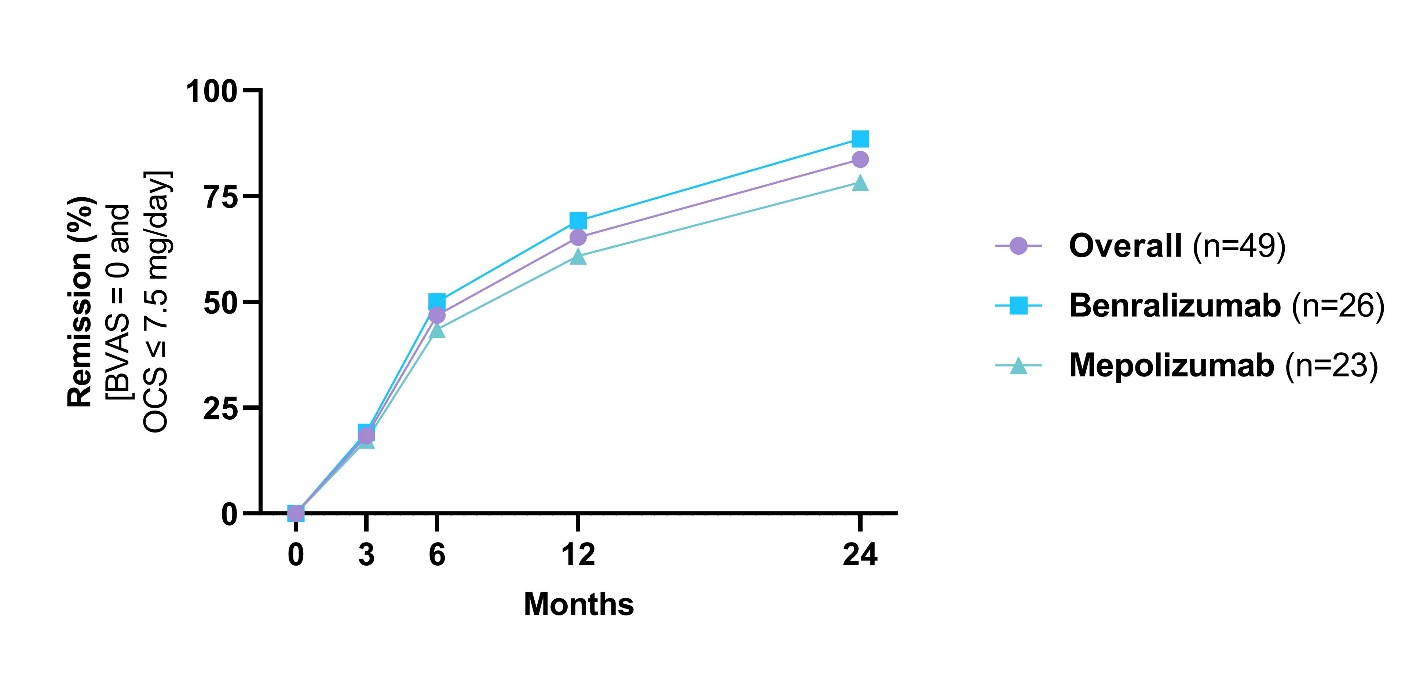
*

*Abbreviations: BVAS, Birmingham vasculitis activity score; OCS, oral corticosteroids (prednisone).*

**Table E3.** Proportion of patients in remission during treatment according to ANCA antibodies

| **Type of response** | **3 months** | | | **6 months** | | | **12 months** | | | **24 months** | | |
| --- | --- | --- | --- | --- | --- | --- | --- | --- | --- | --- | --- | --- |
| **MIRRA/MANDARA remission criteria (BVAS = 0 and OCS ≤ 4 mg/day)** | **ANCA+** | **ANCA-** | ***p-value*** | **ANCA+** | **ANCA-** | ***p-value*** | **ANCA+** | **ANCA-** | ***p-value*** | **ANCA+** | **ANCA-** | ***p-value*** |
| Overall^*^, n (%) | 0 (0) | 2 (5.6) | *0.9999* | 2 (15.4) | 11 (30.6) | *0.4668* | 5 (38.5) | 14 (38.9) | *0.9999* | 7 (53.9) | 21 (58.3) | *0.9999* |
| Benralizumab^†^, n (%) | 0 (0) | 0 (0) | *0.9999* | 0 (0) | 6 (31.6) | *0.1456* | 3 (42.9) | 8 (42.1) | *0.9999* | 5 (71.4) | 13 (68.4) | *0.9999* |
| Mepolizumab^‡^, n (%) | 0 (0) | 2 (11.7) | *0.9999* | 2 (33.3) | 5 (29.4) | *0.9999* | 2 (33.3) | 6 (35.3) | *0.9999* | 2 (33.3) | 8 (47.1) | *0.6600* |
| **EULAR remission criteria (BVAS = 0 and OCS ≤ 7.5 mg/day)** |  |  |  |  |  |  |  |  |  |  |  |  |
| Overall^*^, n (%) | 4 (30.8) | 5 (13.9) | *0.2204* | 6 (46.2) | 17 (47.2) | *0.9999* | 11 (84.6) | 21 (58.3) | *0.1051* | 11 (84.6) | 30 (83.3) | *0.9999* |
| Benralizumab^†^, n (%) | 1 (14.3) | 4 (21.1) | *0.9999* | 2 (28.6) | 11 (57.9) | *0.3783* | 6 (85.7) | 12 (63.2) | *0.3748* | 7 (100) | 16 (84.2) | *0.5396* |
| Mepolizumab^‡^, n (%) | 3 (50) | 1 (5.9) | ***0.0353*** | 4 (66.7) | 6 (35.3) | *0.3413* | 5 (83.3) | 9 (52.9) | *0.3401* | 4 (66.7) | 14 (82.4) | *0.5756* |

*Abbreviations: ANCA, Anti-neutrophil cytoplasmic antibody, BVAS, Birmingham vasculitis activity score; OCS, oral corticosteroids (prednisone).*

^*^ 13 ANCA+ patients, 36 ANCA- patients at baseline

^†^ 7 ANCA+ patients, 19 ANCA- patients at baseline

^‡^ 6 ANCA+ patients, 17 ANCA- patients at baseline

**Table E4.** Baseline characteristics of patients on stable remission from month 12 up to 24 vs non-remitters

|  | **Stable remission from month 12 up to 24 (n=17)** | **No remission (n=32)** | *p****-value^*^*** |
| --- | --- | --- | --- |
| **Female, n (%)** | 11 (64.7) | 20 (62.5) | *0.9999* |
| **BMI, mean (SD)** | 24.5 (3.2) | 25.1 (2.4) | *0.9791* |
| **Age, years, mean (SD)** | 51.1 (11.7) | 49.9 (11.5) | *0.7347* |
| **Age at onset, years, mean (SD)** | 35.6 (12.6) | 37.6 (10.5) | *0.7748* |
| **Disease duration, years, mean (SD)** | 10.9 (13.4) | 14.7 (8.2) | *0.1810* |
| **Smoking status** |  |  |  |
| Smoking history, n (%) | 3 (17.6) | 6 (18.7) | *0.9999* |
| Current smoker, n (%) | 0 (0) | 2 (6.3) | *0.5374* |
| **Patients with positive Skin Prick Tests, n (%)** | 9 (52.9) | 11 (34.4) | *0.2369* |
| **Active disease (BVAS >0), n (%)** | 17 (100) | 28 (87.5) | *0.2837* |
| **BVAS score, median (IQR)** | 8 (4-8) | 6 (4-10) | *0.7474* |
| **VDI, median (IQR)** | 2 (1-2) | 2 (0.8-3.3) | *0.4518* |
| **Five-Factor Score (revisited)** |  |  |  |
| 0, n (%) | 10 (58.8) | 19 (59.4) | *0.9999* |
| 1, n (%) | 4 (25.5) | 12 (37.5) | *0.3601* |
| 2, n (%) | 3 (17.6) | 1 (3.1) | *0.1139* |
| **Organ involvement** |  |  |  |
| Constitutional, n (%) | 3 (17.6) | 8 (25) | *0.7251* |
| Arthropathy, n (%) | 2 (11.8) | 4 (12.5) | *0.9999* |
| Cutaneous, n (%) | 2 (11.8) | 7 (21.9) | *0.4668* |
| ENT, n (%) | 13 (76.5) | 24 (75) | *0.9999* |
| Pulmonary, n (%) | 8 (47) | 21 (65.6) | *0.2369* |
| Cardiac, n (%) | 2 (11.8) | 7 (21.9) | *0.4668* |
| Gastrointestinal, n (%) | 2 (11.8) | 5 (15.6) | *0.9999* |
| Renal, n (%) | 1 (5.9) | 2 (6.3) | *0.9999* |
| Peripheral neuropathy, n (%) | 4 (23.5) | 9 (28.1) | *0.9999* |
| **ANCA antibodies** |  |  |  |
| ANCA positive, n (%) | 4 (23.5) | 9 (28.1) | *0.9999* |
| Perinuclear ANCA, n (%) | 4 (23.5) | 8 (25) | *0.9999* |
| MPO ANCA, n (%) | 1 (5.9) | 1 (3.1) | *0.9999* |
| PR3 ANCA, n (%) | 0 (0) | 1 (3.1) | *0.9999* |
| **Asthma exacerbations/year, median (IQR)** | 4 (2.3-5.8) | 4 (3-5.8) | *0.9086* |
| **ACT, median (IQR)** | 13.5 (9.3-19) | 13.5 (10-16.8) | *0.8707* |
| **FEV_1_, %, median (IQR)** | 73 (54-92.8) | 77.5 (60-88) | *0.6146* |
| **FEV_1_, L, median (IQR)** | 2.1 (1.6-2.5) | 2.3 (2.0-2.6) | *0.2847* |
| **FVC, %, median (IQR)** | 79 (72-114) | 90 (81.8-100) | *0.8611* |
| **FEV_1_/FVC, %, median (IQR)** | 63.5 (57.8-72) | 71 (62-77) | *0.0895* |
| **FEF_25-75_, %, median (IQR)** | 41 (19.5-62) | 41 (25.5-61) | *0.6338* |
| **FeNO, ppb, median (IQR)** | 57.5 (29.5-107) | 62.5 (29.8-129.8) | *0.9527* |
| **Laboratory parameters** |  |  |  |
| Eosinophil counts in peripheral blood, cells/μL median (IQR) | 820 (457-1950) | 752 (471-1504) | *0.9591* |
| Basophil counts in peripheral blood, cells/μL median (IQR) | 70 (21-120) | 53.5 (30-89.3) | *0.5414* |
| Neutrophil counts in peripheral blood, cells/μL median (IQR) | 4370 (3115-5770) | 5160 (3850-6590) | *0.4306* |
| IgE, UI/ml, median (IQR) | 308 (102-585) | 158 (51-780) | *0.3108* |
| **Pharmacologic therapies** |  |  |  |
| High dose ICS-LABA, n (%) | 17 (100) | 32 (100) | *0.9999* |
| LAMA, n (%) | 9 (52.9) | 20 (62.5) | *0.5548* |
| Patients on OCS, n, (%) | 14 (82.3) | 32 (100) | ***0.0369*** |
| OCS, mg/die, median (IQR) | 5 (2.5-10) | 13.8 (6.3-25) | ***0.0005*** |
| **Pantients on DMARDS, n (%)** | 6 (35.3) | 11 (34.4) | *0.9999* |
| Azathioprine, n (%) | 4 (23.5) | 7 (21.9) | *0.9999* |
| Methotrexate, n (%) | 1 (5.9) | 3 (9.4) | *0.9999* |
| Cyclosporine, n (%) | 1 (5.9) | 0 (0) | *0.3469* |
| Rituximab, n (%) | 0 (0) | 1 (3.1) | *0.9999* |
| **Biologic therapy** |  |  |  |
| Benralizumab, n (%) | 11 (64.7) | 15 (46.9) | *0.3675* |
| Mepolizumab 100mg, n (%) | 5 (29.4) | 15 (46.9) | *0.3607* |
| Mepolizumab 300mg, n (%) | 1 (5.9) | 2 (6.3) | *0.9999* |
| Previous anti-IgE/anti-IL-5 mAbs, n (%) | 1 (5.9) | 0 (0) | *0.3469* |

*Abbreviations: ACT, asthma control test; ANCA, anti-neutrophil cytoplasmic antibody; BMI, body mass index; BVAS, Birmingham Vasculitis Activity Score; DMARD, disease-modifying antirheumatic drug; ENT, ear, nose, and throat; FEF_25-75_, forced expiratory flow between 25% and 75% of FVC; FeNO, fractional exhaled nitric oxide; FEV_1_, forced expiratory volume in the 1st second; FVC, forced vital capacity; ICS-LABA, inhaled corticosteroids - long-acting beta-agonist; IgE, immunoglobulin-E; LAMA, long-acting muscarinic antagonist; mAb, monoclonal antibody; MPO, myeloperoxidase; OCS, oral corticosteroids (prednisone equivalent dose); PR3, proteinase 3; VDI, vascular damage index.*

For normally distributed data, values are mean (standard deviation [SD]). For non-normally distributed variables, values are median (interquartile range [IQR]).

Bold entries highlight statistically significant *p*-values.

**Table E5.** Baseline characteristics of patients on remission at month 24 vs non-remitters

|  | **Remission at month 24 (n=28)** | **No remission (n=21)** | ***p-value*** |
| --- | --- | --- | --- |
| **Female, n (%)** | 18 (64.2) | 13 (61.9) | *0.9999* |
| **BMI, mean (SD)** | 25.1 (3.6) | 23.7 (3.4) | *0.1877* |
| **Age, years, mean (SD)** | 48.7 (11.8) | 52.3 (10.9) | *0.3126* |
| **Age at onset, years, mean (SD)** | 35.6 (12.1) | 37.5 (10.2) | *0.3565* |
| **Disease duration, years, mean (SD)** | 11.6 (11.4) | 16.7 (10.2) | *0.1610* |
| **Smoking status** |  |  |  |
| Smoking history, n (%) | 5 (17.8) | 4 (19) | *0.9999* |
| Current smoker, n (%) | 0 (0) | 2 (9.5) | *0.1786* |
| **Patients with positive Skin Prick Tests, n (%)** | 13 (46.4) | 7 (33.3) | *0.3942* |
| **Active disease (BVAS >0), n (%)** | 28 (100) | 17 (81) | ***0.0282*** |
| **BVAS score, median (IQR)** | 6 (4-8) | 8 (4-10) | *0.8907* |
| **VDI, median (IQR)** | 1 (0-2) | 2 (1-4) | *0.1088* |
| **Five-Factor Score (revisited)** |  |  |  |
| 0, n (%) | 16 (57.2) | 13 (61.9) | *0.7768* |
| 1, n (%) | 8 (28.6) | 8 (38.1) | *0.5474* |
| 2, n (%) | 4 (14.3) | 0 (0) | *0.1249* |
| **Organ involvement** |  |  |  |
| Constitutional, n (%) | 4 (14.3) | 7 (33.3) | *0.1687* |
| Arthropathy, n (%) | 3 (10.7) | 3 (14.3) | *0.9999* |
| Cutaneous, n (%) | 4 (14.3) | 5 (23.8) | *0.4698* |
| ENT, n (%) | 21 (75) | 16 (76.2) | *0.9999* |
| Pulmonary, n (%) | 15 (53.6) | 14 (66.7) | *0.3942* |
| Cardiac, n (%) | 3 (10.7) | 6 (28.6) | *0.1460* |
| Gastrointestinal, n (%) | 5 (17.9) | 2 (9.5) | *0.6830* |
| Renal, n (%) | 2 (7.1) | 1 (4.8) | *0.9999* |
| Peripheral neuropathy, n (%) | 4 (14.3) | 9 (42.9) | ***0.0476*** |
| **ANCA antibodies** |  |  |  |
| ANCA positive, n (%) | 7 (25) | 6 (28.6) | *0.9999* |
| Perinuclear ANCA, n (%) | 7 (25) | 5 (23.8) | *0.9999* |
| MPO ANCA, n (%) | 2 (7.1) | 0 (0) | *0.5000* |
| PR3 ANCA, n (%) | 0 (0) | 1 (4.8) | *0.4286* |
| **Asthma exacerbations/year, median (IQR)** | 4 (3-5) | 4 (3-6) | *0.6644* |
| **ACT, median (IQR)** | 15 (11-19) | 13 (9.5-16.5) | *0.2115* |
| **FEV_1_, %, median (IQR)** | 77 (54-85) | 77 (60-92.5) | *0.5194* |
| **FEV_1_, L, median (IQR)** | 2.3 (1.7-2.5) | 2.3 (2.1-2.8) | *0.2757* |
| **FVC, %, median (IQR)** | 90 (73.5-106.8) | 90 (82.5-105) | *0.8341* |
| **FEV_1_/FVC, %, median (IQR)** | 68 (60-73.5) | 71 (62-77) | *0.3097* |
| **FEF_25-75_, %, median (IQR)** | 43.8 (25.8-59.3) | 34 (23-63) | *0.7755* |
| **FeNO, ppb, median (IQR)** | 51 (30-82) | 75 (31.5-132.5) | *0.4882* |
| **Laboratory parameters** |  |  |  |
| Eosinophil counts in peripheral blood, cells/μL median (IQR) | 1005 (511-2295) | 610 (434-1245) | ***0.0189*** |
| Basophil counts in peripheral blood, cells/μL median (IQR) | 70 (40-105) | 30 (29-89) | *0.1417* |
| Neutrophil counts in peripheral blood, cells/μL median (IQR) | 4600 (3000-6760) | 4810 (4062-5986) | *0.5197* |
| IgE, UI/ml, median (IQR) | 213 (72-624) | 113 (46-407) | *0.5080* |
| **Pharmacologic therapies** |  |  |  |
| High dose ICS-LABA, n (%) | 28 (100) | 21 (100) | *0.9999* |
| LAMA, n (%) | 14 (50) | 15 (71.4) | *0.1541* |
| Patients on OCS, n, (%) | 25 (89.3) | 21 (100) | *0.2500* |
| OCS, mg/die, median (IQR) | 5 (2.5-12.5) | 15 (10-25) | ***0.0043*** |
| **Pantients on DMARDS, n (%)** | 10 (35.7) | 7 (33.3) | *0.9999* |
| Azathioprine, n (%) | 7 (25) | 4 (19) | *0.7369* |
| Methotrexate, n (%) | 2 (7.1) | 2 (9.5) | *0.9999* |
| Cyclosporine, n (%) | 1 (3.6) | 0 (0) | *0.9999* |
| Rituximab, n (%) | 0 (0) | 1 (4.8) | *0.4286* |
| **Biologic therapy** |  |  |  |
| Benralizumab, n (%) | 18 (64.3) | 8 (38.1) | *0.0882* |
| Mepolizumab 100mg, n (%) | 8 (28.6) | 12 (42.9) | *0.0771* |
| Mepolizumab 300mg, n (%) | 2 (7.1) | 1 (4.8) | *0.9999* |
| Previous anti-IgE/anti-IL-5 mAbs, n (%) | 1 (3.6) | 0 (0) | *0.9999* |

*Abbreviations: ACT, asthma control test; ANCA, anti-neutrophil cytoplasmic antibody; BMI, body mass index; BVAS, Birmingham Vasculitis Activity Score; DMARD, disease-modifying antirheumatic drug; ENT, ear, nose, and throat; FEF_25-75_, forced expiratory flow between 25% and 75% of FVC; FeNO, fractional exhaled nitric oxide; FEV_1_, forced expiratory volume in the 1st second; FVC, forced vital capacity; ICS-LABA, inhaled corticosteroids - long-acting beta-agonist; IgE, immunoglobulin-E; LAMA, long-acting muscarinic antagonist; mAb, monoclonal antibody; MPO, myeloperoxidase; OCS, oral corticosteroids (prednisone equivalent dose); PR3, proteinase 3; VDI, vascular damage index.*

For normally distributed data, values are mean (standard deviation [SD]). For non-normally distributed variables, values are median (interquartile range [IQR]).

Bold entries highlight statistically significant *p*-values.

**Table E6.** DMARDs discontinuation during treatment

|  | **Baseline** | **3 months** | ***p-value*** | **6 months** | ***p-value*** | **12 months** | ***p-value*** | **24 months** | ***p-value*** |
| --- | --- | --- | --- | --- | --- | --- | --- | --- | --- |
| **Pantients on DMARDs** |  |  |  |  |  |  |  |  |  |
| Overall (n=49), n (%) | 17 (34.7) | 12 (24.5) | *0.0736* | 7 (14.3) | ***0.0044*** | 5 (10.2) | ***0.0015*** | 2 (4.1) | ***0.0003*** |
| Benralizumab (n=26), n (%) | 11 (42.3) | 7 (26.9) | *0.1336* | 3 (11.5) | ***0.0133*** | 1 (3.8) | ***0.0044*** | 0 (0) | ***0.0026*** |
| Mepolizumab (n=23), n (%) | 6 (26.1) | 5 (21.8) | *0.9999* | 4 (17.4) | *0.4795* | 4 (17.4) | *0.4795* | 2 (8.7) | *0.1336* |
| **Azathioprine** |  |  |  |  |  |  |  |  |  |
| Overall, n (%) | 11 (22.4) | 8 (30.8) | *0.2482* | 5 (19.2) | ***0.0412*** | 4 (15.4) | ***0.0233*** | 1 (2) | ***0.0044*** |
| Benralizumab, n (%) | 7 (26.9) | 4 (15.4) | *0.2482* | 2 (7.7) | *0.0736* | 1 (3.8) | ***0.0412*** | 0 (0) | ***0.0233*** |
| Mepolizumab, n (%) | 4 (17.4) | 4 (17.4) | *0.9999* | 3 (13) | *0.9999* | 3 (13) | *0.9999* | 1 (4.3) | *0.2482* |
| **Methotrexate** |  |  |  |  |  |  |  |  |  |
| Overall, n (%) | 4 (8.2) | 4 (8.2) | *0.9999* | 2 (4.1) | *0.4795* | 1 (2) | *0.2482* | 1 (2) | *0.2482* |
| Benralizumab, n (%) | 3 (11.5) | 3 (11.5) | *0.9999* | 1 (3.8) | *0.4795* | 0 (0) | *0.2482* | 0 (0) | *0.2482* |
| Mepolizumab, n (%) | 1 (4.3) | 1 (4.3) | *0.9999* | 1 (4.3) | *0.9999* | 1 (4.3) | *0.9999* | 1 (4.3) | *0.9999* |
| **Cyclosporine** |  |  |  |  |  |  |  |  |  |
| Overall, n (%) | 1 (2) | 0 (0) | *0.9999* | 0 (0) | *0.9999* | 0 (0) | *0.9999* | 0 (0) | *0.9999* |
| Benralizumab, n (%) | 0 (0) | 0 (0) | *0.9999* | 0 (0) | *0.9999* | 0 (0) | *0.9999* | 0 (0) | *0.9999* |
| Mepolizumab, n (%) | 1 (4.3) | 0 (0) | *0.9999* | 0 (0) | *0.9999* | 0 (0) | *0.9999* | 0 (0) | *0.9999* |
| **Rituximab** |  |  |  |  |  |  |  |  |  |
| Overall, n (%) | 1 (2) | 0 (0) | *0.9999* | 0 (0) | *0.9999* | 0 (0) | *0.9999* | 0 (0) | *0.9999* |
| Benralizumab, n (%) | 1 (3.8) | 0 (0) | *0.9999* | 0 (0) | *0.9999* | 0 (0) | *0.9999* | 0 (0) | *0.9999* |
| Mepolizumab, n (%) | 0 (0) | 0 (0) | *0.9999* | 0 (0) | *0.9999* | 0 (0) | *0.9999* | 0 (0) | *0.9999* |

*Abbreviation: DMARD, disease-modifying antirheumatic drug.*

Changes in DMARDs regimen during follow-ups were assessed using the McNemar test.

Bold entries highlight statistically significant *p*-values.

**Table E7.** Baseline characteristics of patients on DMARDs vs no DMARDs

|  | **Patients on DMARDs (n=17)** | **No DMARDs (n=32)** | ***p-value*** |
| --- | --- | --- | --- |
| **Female, n (%)** | 11 (64.7) | 20 (62.5) | *0.9999* |
| **BMI, mean (SD)** | 24.6 (2.9) | 24.4 (3.8) | *0.8575* |
| **Age, years, mean (SD)** | 51.9 (14) | 49.4 (10) | *0.4832* |
| **Age at onset, years, mean (SD)** | 36.1 (13.2) | 37.4 (10.1) | *0.7169* |
| **Disease duration, years, mean (SD)** | 14.4 (15.1) | 12 (7.6) | *0.4692* |
| **Smoking status** |  |  |  |
| Smoking history, n (%) | 4 (23.5) | 5 (15.6) | *0.7001* |
| Current smoker, n (%) | 0 (0) | 2 (6.3) | *0.5374* |
| **Patients with positive Skin Prick Tests, n (%)** | 6 (35.3) | 14 (43.8) | *0.7611* |
| **Active disease (BVAS >0), n (%)** | 17 (100) | 28 (87.5) | *0.2837* |
| **BVAS score, median (IQR)** | 6 (4-9) | 6 (4-10) | *0.7474* |
| **VDI, median (IQR)** | 3 (1.8-4.5) | 1 (0-2) | ***0.0268*** |
| **Five-Factor Score (revisited)** |  |  |  |
| 0, n (%) | 7 (41.2) | 22 (68.8) | *0.0753* |
| 1, n (%) | 7 (41.2) | 9 (28.1) | *0.5231* |
| 2, n (%) | 3 (17.6) | 1 (3.1) | *0.1139* |
| **Organ involvement** |  |  |  |
| Constitutional, n (%) | 5 (29.4) | 6 (18.8) | *0.4796* |
| Arthropathy, n (%) | 4 (23.5) | 2 (6.3) | *0.1643* |
| Cutaneous, n (%) | 3 (17.7) | 6 (18.8) | *0.9999* |
| ENT, n (%) | 13 (76.5) | 24 (75) | *0.9999* |
| Pulmonary, n (%) | 11 (64.7) | 18 (56.3) | *0.7611* |
| Cardiac, n (%) | 7 (41.2) | 2 (6.3) | ***0.0051*** |
| Gastrointestinal, n (%) | 3 (17.6) | 4 (12.5) | *0.6812* |
| Renal, n (%) | 2 (11.8) | 1 (3.1) | *0.2731* |
| Peripheral neuropathy, n (%) | 5 (29.4) | 8 (25) | *0.7458* |
| **ANCA antibodies** |  |  |  |
| ANCA positive, n (%) | 6 (35.3) | 7 (21.9) | *0.3309* |
| Perinuclear ANCA, n (%) | 6 (35.3) | 6 (18.8) | *0.2962* |
| MPO ANCA, n (%) | 1 (5.9) | 1 (3.1) | *0.9999* |
| PR3 ANCA, n (%) | 0 (0) | 1 (3.1) | *0.9999* |
| **Asthma exacerbations/year, median (IQR)** | 4.5 (3-5.8) | 4 (3-5.8) | *0.5941* |
| **ACT, median (IQR)** | 15 (10.3-19) | 13 (10-17) | *0.6682* |
| **FEV_1_, %, median (IQR)** | 77 (63.3-88.8) | 77 (59-93.3) | *0.9612* |
| **FEV_1_, L, median (IQR)** | 2.3 (1.8-2.6) | 2.3 (1.8-2.6) | *0.9784* |
| **FVC, %, median (IQR)** | 97 (75-106) | 88 (75-106) | *0.4132* |
| **FEV_1_/FVC, %, median (IQR)** | 66 (60-72) | 72 (60.5-78) | *0.2089* |
| **FEF_25-75_, %, median (IQR)** | 41 (25.5-58.5) | 41 (22-62.3) | *0.8995* |
| **FeNO, ppb, median (IQR)** | 101 (28.8-248) | 57 (30.8-92.8) | *0.3043* |
| **Laboratory parameters** |  |  |  |
| Eosinophil counts in peripheral blood, cells/μL median (IQR) | 630 (485-1603) | 845 (442-1585) | *0.7193* |
| Basophil counts in peripheral blood, cells/μL median (IQR) | 30 (25.5-65) | 70 (44.8-115) | *0.0764* |
| Neutrophil counts in peripheral blood, cells/μL median (IQR) | 4525 (2995-6815) | 4950 (3950-5830) | *0.9552* |
| IgE, UI/ml, median (IQR) | 80 (42-780) | 213 (99-214) | *0.4824* |
| **Pharmacologic therapies** |  |  |  |
| High dose ICS-LABA, n (%) | 17 (100) | 32 (100) | *0.9999* |
| LAMA, n (%) | 9 (52.9) | 20 (62.5) | *0.5548* |
| Patients on OCS, n, (%) | 17 (100) | 29 (90.6) | *0.5423* |
| OCS, mg/die, median (IQR) | 15 (5-23.8) | 10 (5-18.3) | *0.3934* |
| **Pantients on DMARDS, n (%)** | 17 (100) | 0 (0) | *n/a* |
| Azathioprine, n (%) | 11 (22.4) | 0 (0) | *n/a* |
| Methotrexate, n (%) | 4 (8.2) | 0 (0) | *n/a* |
| Cyclosporine, n (%) | 1 (2) | 0 (0) | *n/a* |
| Rituximab, n (%) | 1 (2) | 0 (0) | *n/a* |
| **Biologic therapy** |  |  |  |
| Benralizumab, n (%) | 11 (64.7) | 15 (46.9) | *0.3675* |
| Mepolizumab 100mg, n (%) | 6 (35.3) | 15 (46.9) | *0.5490* |
| Mepolizumab 300mg, n (%) | 1 (5.9) | 2 (6.3) | *0.9999* |
| Previous anti-IgE/anti-IL-5 mAbs, n (%) | 0 (0) | 1 (3.1) | *0.9999* |

*Abbreviations: ACT, asthma control test; ANCA, anti-neutrophil cytoplasmic antibody; BMI, body mass index; BVAS, Birmingham Vasculitis Activity Score; DMARD, disease-modifying antirheumatic drug; ENT, ear, nose, and throat; FEF_25-75_, forced expiratory flow between 25% and 75% of FVC; FeNO, fractional exhaled nitric oxide; FEV_1_, forced expiratory volume in the 1st second; FVC, forced vital capacity; ICS-LABA, inhaled corticosteroids - long-acting beta-agonist; IgE, immunoglobulin-E; LAMA, long-acting muscarinic antagonist; mAb, monoclonal antibody; MPO, myeloperoxidase; OCS, oral corticosteroids (prednisone equivalent dose); PR3, proteinase 3; VDI, vascular damage index.*

For normally distributed data, values are mean (standard deviation [SD]). For non-normally distributed variables, values are median (interquartile range [IQR]).

Bold entries highlight statistically significant *p*-values.

**Table E8.** Adverse events

| **Event** | **Overall (n=49)** | **Benralizumab (n=26)** | **Mepolizumab (n=23)** |
| --- | --- | --- | --- |
| **Total adverse events, n (%)** | 4 (8.2) | 2 (7.7) | 2 (8.7) |
| **Mild-moderate adverse events, n (%)** | 3 (6.1) | 1 (3.8) | 2 (8.7) |
| Arrhythmia, n (%) | 1 (2) | 0 (0) | 1 (4.3) |
| Urticaria, n (%) | 1 (2) | 0 (0) | 1 (4.3) |
| Otitis, n (%) | 1 (2) | 1 (3.8) | 0 (0) |
| **Serious adverse events, n (%)** | 1 (2) | 1 (3.8) | 0 (0) |
| Pneumonia, n (%) | 0 (0) | 1 (3.8) | 0 (0) |
| **Adverse event requiring treatment discontinuation, n (%)** | 0 (0) | 0 (0) | 0 (0) |
